# Supplementary material for: Progressive, Qualitative, and Quantitative Alterations in HDL Lipidome from Healthy Subjects to Patients with Prediabetes and Type 2 Diabetes
Source: Metabolites. 2022 Jul 25;12(8):683. doi: 10.3390/metabo12080683 (PMC9331261; doi:10.3390/metabo12080683)

Supplementary Data

Progressive, qualitative and quantitative alterations in HDL lipidome from healthy subjects to patients with Prediabetes and Type 2 Diabetes

Christina E. Kostara[1], Kiriaki S. Karakitsou[1], Matilda Florentin[2], Eleni T. Bairaktari[1] and Vasilis Tsimihodimos[2,*]

[1] Laboratory of Clinical Chemistry, Faculty of Medicine, University of Ioannina, Ioannina, Greece; chkostara@gmail.com, ebairakt@uoi.gr
[2] Department of Internal Medicine, Faculty of Medicine, University of Ioannina, Ioannina, Greece; vtsimi@uoi.gr
*Correspondence: vtsimi@uoi.gr; Tel.: +302651007362

Multivariate statistical analysis

OPLS-DA analysis of the HDL lipidomic data was performed for the three pair-wise comparisons (normoglycemic group vs prediabetes, prediabetes vs T2D and normoglycemic group vs T2D). After reducing data dimensions to the principal components, the score plots for the three comparisons showed clustering into separate groups with good goodness of fit and predictive power (Table S1), and minimal overlap (Figure S1a, c, e).

Prediabetes vs normoglycemic group: The analysis of the loading coefficient plot of the resulting model (Figure S1b) that means the relative significance of each constituent in the groups' separation, revealed that the determining lipid constituents were the lower levels of choline phospholipids, unsaturated fatty acids, ω-3 fatty acids and cholesterol esters in prediabetes and the higher levels of saturated fatty acids and core triglycerides.

Type 2 Diabetes (T2D) vs Prediabetes: The determining lipid constituents in the separation are depicted in Figure S1d. It is notable that in this model the determining constituents found previously in the Prediabetes vs normoglycemic model were further deteriorated towards the same direction as in T2D patients, e.g., choline phospholipids and cholesterol esters were further reduced; saturated fatty acids and core triglycerides were further increased.

T2D vs Normoglycemic group: Finally, the same lipid alterations in HDL particles were observed in the T2D patients vs. normoglycemic group model (Figure S1f).

Table S1. Parameters of the OPLS-DA models

Statistical Model	R^2X	R^2Y	Q^2Y	P value
Normoglycemic group vs Prediabetes	0.592	0.621	0.394	<0.0001
Prediabetes vs T2D	0.568	0.64	0.456	<0.0001
Normoglycemic group vs T2D	0.563	0.725	0.604	<0.001

Figure S1: OPLS-DA score plot of the HDL lipidomic data from: **(a)** 40 normoglycemic individuals (black triangles) and 40 patients with prediabetes (red triangles); (b) the corresponding regression coefficient plot; (c) 40 patients with prediabetes (red triangles) and 40 patients with type 2 diabetes (T2D) (gray triangles) (d) the corresponding regression coefficient plot; (e) 40 normoglycemic individuals (black triangles) and 40 patients with type 2 diabetes (T2D) (gray triangles) and (f) the corresponding regression coefficient plot. Abbreviations: **CE**, Cholesterol Esters; **LA**, Linoleic acid; **PC**, Phosphatidylcholine; **PLs**, Phospholipids; **PUFA**, Polyunsaturated fatty acids; **SFA**, Saturated fatty acids; **SM**, Sphingomyelin; **TG**, Triglycerides**, UFA**, Unsaturated fatty acids, and ***ω*-3 FA**, total omega-3 fatty acids

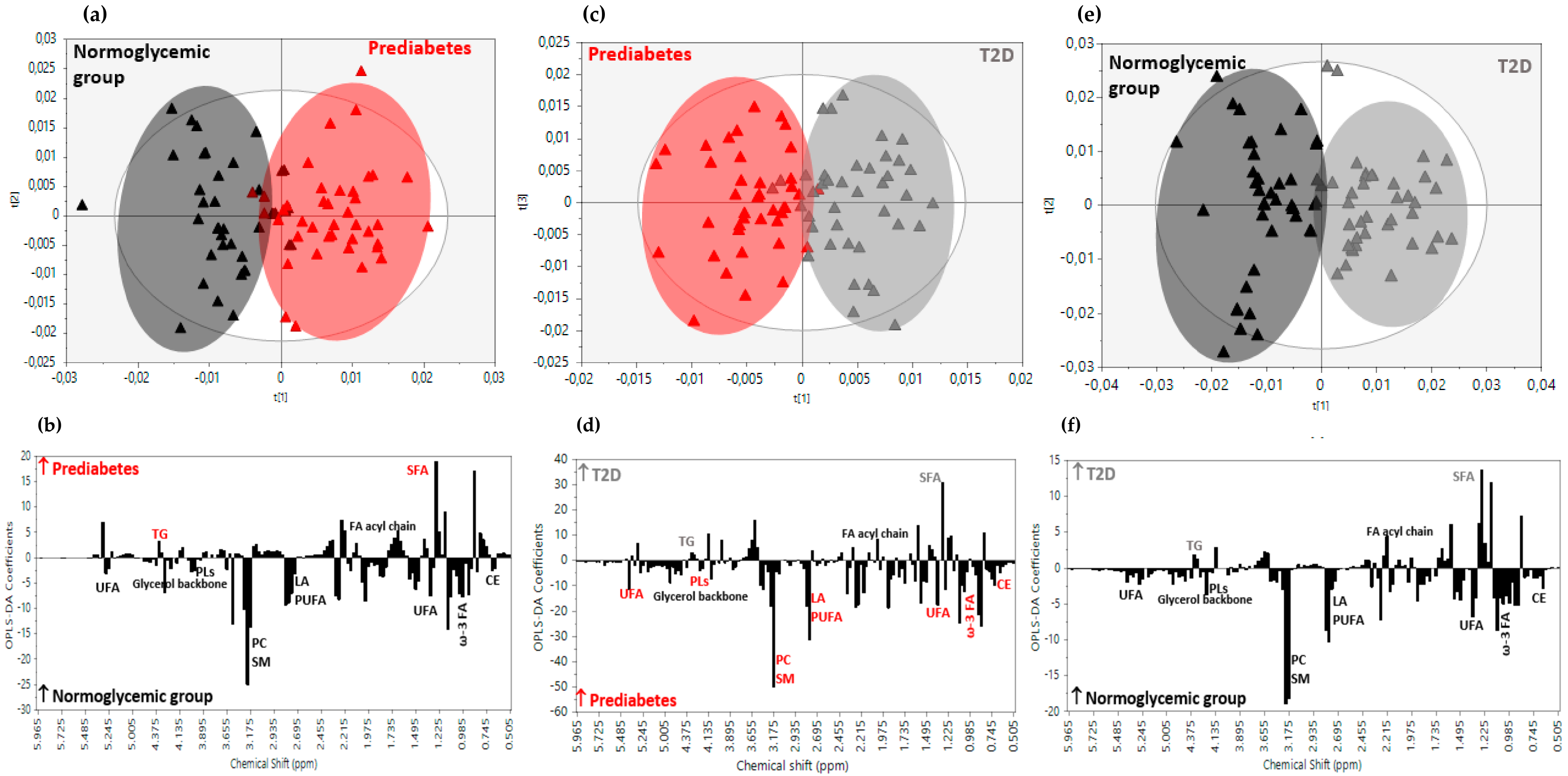

Supplement: Supplementary file 1 [file metabolites-12-00683-s001.zip › metabolites-1792184-supplementary.pdf]
